# Management of Musculoskeletal Manifestations in Inflammatory Bowel Disease

**DOI:** 10.1155/2015/387891

**Published:** 2015-06-10

**Authors:** Tejas Sheth, C. S. Pitchumoni, Kiron M. Das

**Affiliations:** ^1^Department of Rheumatology, Albert Einstein College of Medicine, Bronx, NY, USA; ^2^Department of Gastroenterology, Rutgers-St. Peter's University Hospital, New Brunswick, NJ, USA; ^3^Division of Gastroenterology and Hepatology, Crohn's and Colitis Center of NJ, Rutgers-Robert Wood Johnson Medical School, Clinical Academic Building, 125 Paterson Street, Suite 5100B, New Brunswick, NJ 08901-1962, USA

## Abstract

Musculoskeletal manifestations are the most common extraintestinal manifestations in inflammatory bowel diseases. Some appendicular manifestations are independent of gut inflammation and are treated with standard anti-inflammatory strategies. On the other hand, axial involvement is linked to gut inflammatory activity; hence, there is a considerable amount of treatment overlap. Biological therapies have revolutionized management of inflammatory bowel diseases as well as of associated articular manifestations. Newer mechanisms driving gut associated arthropathy have surfaced in the past decade and have enhanced our interests in novel treatment targets. Introduction of biosimilar molecules is expected in the US market in the near future and will provide an opportunity for considerable cost savings on healthcare. A multidisciplinary approach involving a gastroenterologist, rheumatologist, and physical therapist is ideal for these patients.

## 1. Introduction

Inflammatory bowel diseases (IBD), Crohn's disease (CD), and ulcerative colitis (UC) are chronic inflammatory disorders of multiple organ systems, primarily involving gut, with chronic relapsing and remitting course. As a result of underlying immune dysregulation, up to 40% of cases are associated with extraintestinal manifestations (EIM). EIM can be classified in to 3 different categories based on their relationship with gut inflammation (Table 1); the principles of management can be widely different across these groups.

Musculoskeletal manifestations (MSM) are the most commonly observed EIM in patients with IBD. Distinct cell-mediated and humoral immunopathophysiological mechanisms have been identified that link gut and synovial inflammation under “the gut-synovial axis.” The arthropathies in IBD patients can affect peripheral joints (type 1 and type 2 peripheral arthritides, arthralgia without arthritis, enthesitis, or dactylitis) or axial skeleton (inflammatory back pain, isolated sacroiliitis, or ankylosing spondylitis) or both. In addition, metabolic bone diseases and chronic widespread pain syndromes are also frequently encountered in IBD patients. The salient clinical features and observed prevalence rates of various MSM in IBD are summarized in Table 2.

This paper reviews the management and currently available treatment options for MSM in IBD and is an addition to our previous review addressing the immunopathophysiology and clinical features of the same [1].

## 2. Peripheral Arthritis

Two distinct types of peripheral arthritides have been described in association with IBD. Type 1 peripheral arthritis, an asymmetric oligoarthritis, is more common and is usually associated with flares of IBD. In contrast, type 2 peripheral arthritis presenting as progressive symmetrical polyarthritis is independent of gut inflammation. The latter is more aggressive and may cause erosions. Peripheral arthritis presents as pain and swelling in one or more joints with or without morning stiffness. On physical examination (PE), the affected joints may show signs of inflammation-warmth, erythema, tenderness to palpation, and synovitis with or without effusion.

Serum inflammatory markers like ESR and C-reactive protein (CRP) reflect bowel inflammation and may be useful in type 2 peripheral arthritis to monitor the disease activity and clinical response. These markers, however, should not be used as surrogates of physical examination to establish the diagnosis. Aspiration of synovial fluid is indicated in acute monoarthritis to rule out infectious or crystal induced arthropathy. It is important to remember that septic arthritis may have atypical presentations in patients with IBD receiving immunosuppressive therapy and the treating physician should execute a high index of suspicion.

*Management*. The goals of treatment of peripheral arthritis are to reduce pain, swelling, and stiffness and to preserve functionality. Type 1 peripheral arthritis is self-limiting and usually resolves with treatment of underlying IBD flare. Management of type 2 peripheral arthritis usually requires a more aggressive approach.

**Figure 1:**
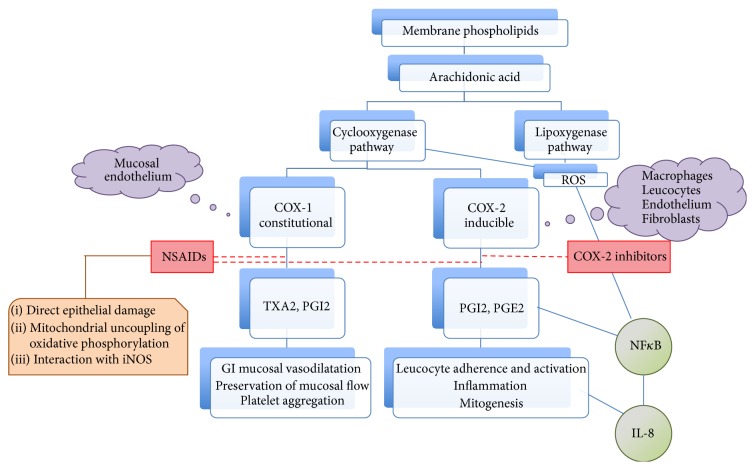
Effects of NSAIDs and selective COX-2 inhibitors. Phospholipase A2 acts on the membrane phospholipids to yield arachidonic acid (AA). AA is metabolized by either cyclooxygenase (COX) or lipoxygenase (LOX) pathway. Two unique COX isoenzymes convert AA into prostaglandin endoperoxides. COX-1 is expressed constitutively in most cells, including GI mucosal cells. In contrast, COX-2 is expressed by inflammatory cells in response to a variety of stimuli including microbial products and cytokines. COX-1 generates prostanoids responsible for “housekeeping” function; they help with vasodilatation, preserving mucosal flow, and induction of platelet aggregation in response to vascular injury to prevent blood loss. COX-2 induction plays a part in leucocyte activation, adherence, and angiogenesis through effects on NF*κ*B and IL8. Reactive oxygen species generated due to enzymatic activities of COX and LOX also stimulate NF*κ*B and perpetuate the cycle. Inhibition of COX-2 prevents this inflammatory cascade and is responsible for clinical effects of NSAIDs and selective COX-2 inhibitors. NSAIDs, in addition, also block COX-1 pathway leading to mucosal injury, vasoconstriction, mucosal ischemia, and increased vascular permeability. NSAIDs may also lead to direct epithelial damage and mitochondrial uncoupling of oxidative phosphorylation. Dashed red line indicates enzymatic inhibition.

Acetaminophen, nonsteroidal anti-inflammatory drugs (NSAIDs), or selective cyclooxygenase- (COX-) 2 inhibitors reduce the inflammation and provide symptomatic relief. The safety of these agents in patients with IBD is a matter of debate. The literature regarding the risk of IBD relapse due to NSAIDs has been equivocal. NSAIDs have toxic effects in the gastrointestinal (GI) tract and theoretically can exacerbate IBD by a number of mechanisms: inhibition of COX pathway, shunting of leukotriene, and alteration of NF*κ*B and IL8 activity (Figure 1) are amongst a few [2, 3]. Decrease in the levels of prostaglandin E2 subsequent to blockage of COX-1 and COX-2 by nonselective NSAIDs appears to be an index event leading to exacerbation of colitis [4]. Although clinical evidences to establish a cause-effect association between NSAIDs and IBD flares are inconclusive, overall literature suggests NSAIDs predispose flare-up and cause delay in therapeutic response [2–4].

In contrast, COX-2 inhibitors may be safe and beneficial in most patients with IBD [5]. Etoricoxib, in doses of 60–120 mg daily for up to 3 months, was not associated with exacerbation in UC and CD patients [6]. Celecoxib, another COX-2 inhibitor, is considered to be even more “gut-friendly.” Experimental studies have shown that nonselective NSAIDs and Etoricoxib can induce enteropathy through a topical action, whereas Celecoxib lacks these detrimental actions and causes a lower degree of intestinal damage [7]. Similar findings have been observed in human studies [8, 9]. Patients with UC with a present or past history of nonspecific arthritis, arthralgia, or other conditions amenable to NSAIDs, when treated with Celecoxib for up to 14 days, showed no greater relapse rate as compared to placebo [10]. Treatment with COX-2 inhibitors, at least for the short term, appears to cause no increased risk of flare of quiescent ulcerative colitis. No randomized, blinded trials with COX-2 inhibitors have been done in CD. However, in a study of 33 patients with IBD (majority were CD), treatment with COX-2 inhibitors was found to be associated with a high incidence of exacerbation of the underlying IBD and GI related complications [11]. To conclude, a meta-analysis found no statistically significant differences in IBD relapse rates between COX-2 inhibitors and placebo [12]. However, it is premature to predict any long-term consequences on the basis of limited data availability. The possible GI adverse effects profile of NSAIDs and COX-2 inhibitors is extensive and beyond the scope of this review. The current recommendations by ACG identify NSAIDs as a recognized risk factor exacerbating IBD [13].

Injection of corticosteroids into the affected joint is a consideration in cases of monoarthritis or when patient cannot tolerate medications due to systemic side effects. Septic arthritis needs to be ruled out before contemplating intra-articular steroid therapy.

**Figure 2:**
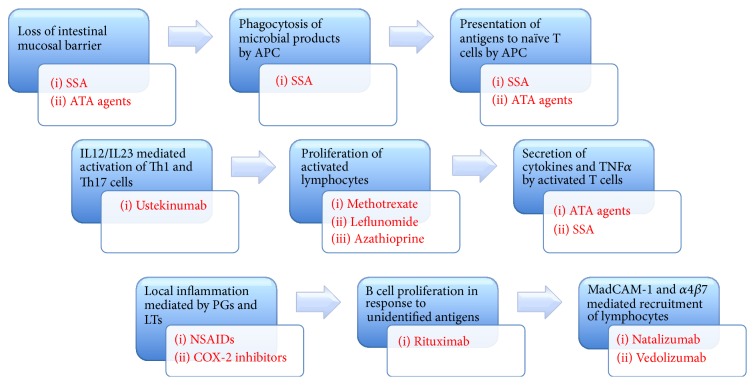
The mechanisms of action of various drugs on the gut-synovial axis. SSA and ATA agents restore increased permeability of the inflamed gut mucosa, thus preventing exposure of PAMPs (pathogen associated molecular patterns) and DAMPs (danger associated molecular patterns) to mucosal APCs. Moreover, SSA directly inhibits phagocytosis and intracellular processing of the ingested antigens, thus reducing MHC restricted presentation of processed peptides to naïve T cells, T cells activation, and subsequent release of inflammatory cytokines. ATA agents downregulate T cell clonal proliferation in gut and in synovium by inhibiting release of proinflammatory cytokines and promoting apoptosis of activated T cells. ATA agents also induce regulatory T cell phenotypes and directly neutralize soluble and membrane bound TNF*α* molecules. Antimetabolites (azathioprine, methotrexate, and leflunomide) inhibit pyrimidine synthesis, thus preventing clonal proliferation of activated T lymphocytes. Rituximab prevents secretion of antibodies directed against autoreactive antigens present in synovium by inhibiting B cells via CD20 antagonism. Ustekinumab directly inhibits IL12 and IL23 mediated Th1 and Th17 cell responses both in gut and in synovium. NSAIDs inhibit production of prostaglandins and mitigate local inflammation. Interaction between *α*4*β*7 integrin expressed on the surface of activated lymphocytes and MadCAM-1 expressed on high endothelial venules is inhibited by antibodies designed against integrin molecules—Natalizumab and vedolizumab (more gut specific). Most of these agents execute similar cellular and anti-inflammatory effects both in gut and in synovium, thus providing a significant treatment overlap. SSA: sulfasalazine, ATA: anti-TNF*α*, NSAIDs: nonsteroidal anti-inflammatory drugs, APCs: antigen presenting cells, MadCAM-1: mucosal addressin cell adhesion molecule-1, PG: prostaglandin, and LT: leukotriene.

Sulfasalazine (SASP) or salicyl-azo-sulphapyridine, the first drug used in UC in 1940s, is found to be effective in treating peripheral arthritis in IBD patients [14]. Particularly in UC, it is most useful in cases of mild to moderate arthritis. The effectiveness of sulfasalazine in arthritis may be related to its ability to suppress synthesis of prostaglandins and leukotrienes. It has been shown to normalize permeability of inflamed gut mucosa, thus preventing exposure of DAMPs (danger-associated molecular patterns) to intestinal APCs (antigen presenting cells) [1]. In addition, sulfasalazine directly inhibits macrophages and polymorphonuclear leukocytes leading to inhibition of (a) phagocytosis, (b) chemotaxis, (c) release of proinflammatory cytokines such as IL1, IL2, and TNF*α*, and (d) the NF*κ*B pathway (Figure 2) [15–17]. All these effects may halt the inflammatory cascade of the gut-synovial axis and may prevent synovial homing of the primed mediator cells, leading to clinical effectiveness [1]. These mechanisms, however, do not explain its limited effectiveness in pure axial involvement. The initial dose of sulfasalazine is 500 mg twice daily for 2-3 weeks with a gradual increase to the maximum of 3000 mg daily in divided doses. This slow induction of treatment with SASP helps tolerance to the drug and avoids side effects related to sulphapyridine component of SASP, which seems to be the effective moiety for arthritis [14]. The other moiety of SASP is the 5-aminosalicylate or 5-ASA; it is identified as responsible molecule for its anti-inflammatory effects topically in the colon and small intestine in patients with IBD. 5-ASA is very little absorbed from the gut and hence does not have much anti-inflammatory effect for arthritis [14, 18].

Methotrexate (MTX), an antimetabolite, is the first line choice for disease modification therapy in rheumatoid arthritis (RA) patients. MTX mediates its anti-inflammatory and antiproliferative effects via several mechanisms: inhibition of dihydrofolate reductase (DHFR) inhibition, decreased pyrimidine synthesis due to inhibition of thymidylate synthetase, and increased adenosine. MTX has been shown to induce and maintain remissions in CD patients [19, 20]. However, there are no randomized controlled trials evaluating efficacy of immunosuppression with MTX, azathioprine, or leflunomide in peripheral arthritis in IBD patients. Based on their established role in treatment of RA and anecdotal reports of their effectiveness in IBD associated arthritis, they can be tried in the event of failure of other therapies; their use in peripheral arthritis in IBD population is more empirical than evidence based. MTX is used in doses of 10 to 25 mg once week, administered orally or subcutaneously (SC). GI intolerance is the most common adverse effect associated with its use; SC injections are associated with less GI toxicities and higher bioavailability. Other side effects of MTX include alopecia, oral ulcerations (may clinically mimic CD oral ulcers), and bone marrow suppression (less with once a week dosing). All the patients receiving MTX should be on daily folic acid supplementations.

The role of anti-TNF*α* agents (ATA) in management of peripheral arthritis has been described later under biological treatment.

## 3. Enthesitis

Enthesitis, a frequent appendicular manifestation of spondyloarthropathy (SpA) seen in IBD patients, refers to inflammation at the tendon attachment site to the bone [1]. Clinically it presents as pain and swelling at the insertion site of Achilles tendon on the heel, plantar fascia insertion site on calcaneus, or the insertion site of patellar tendon on the knee. The clinical assessment of enthesitis in patients with SpA is an important outcome measure; enthesitis indices such as SPARCC (Spondyloarthritis Research Consortium of Canada), Mander index, a modified Mander index, the Maastricht AS Enthesitis Score, the Leeds Enthesitis Index, and the Major Enthesitis Index have been validated to reflect disease activity in SpA populations [21, 22].

The diagnosis can be made by physical examination; imaging is not necessary in average cases. Local radiography may show erosive lesions with spur formation and ossification of entheses at advanced stages. Musculoskeletal ultrasound (MSU) is being increasingly used for diagnostic and posttreatment evaluation of enthesitis. MSU features suggestive of enthesitis include tendon edema, peritendinitis, tendon calcifications, increased Doppler power signal, bony entheseal erosions, and adjacent bone marrow edema [23]. Improvement in the MSU technique by adding B mode power Doppler or contrast-enhanced ultrasound improves the diagnostic accuracy of MSU for assessment of enthesitis, making it a highly specific diagnostic modality [24, 25].

*Management*. The mainstay of treatment of enthesitis is NSAIDs and COX-2. In severe and resistant cases, treatment with ATA agents can be initiated. Etanercept has been shown to be effective in refractory heel enthesitis [26]. In one study adalimumab, given for 12 weeks, has been shown to be effective in reducing signs and symptoms of enthesitis related arthritis in children, with the clinical benefit sustained up to 52 weeks [27]. Ultrasound guided local injection of etanercept has been tried with success [28]. Interestingly, AS patients with enthesitis are less likely to achieve partial remission with standard ATA therapies [29].

## 4. Dactylitis

Dactylitis refers to pain and swelling of an entire digit, seen in up to 4% of IBD patients, and is associated with SpA [1]. The diagnosis is established by clinical examination. The burden of dactylitis is graded clinically by the number of affected digits and by physician-rated severity assessment. The Leeds dactylitis instrument (LDI) is an objective tool designed to grade the dactylitis severity based on the median difference in digital circumference between dactylitic digits and control digits [30]. MSU and MRI can be useful for further evaluation of dactylitis although it adds little to clinical examination to aid the diagnosis [31]. MSU features of dactylitis include flexor tendon tenosynovitis and joint synovitis with or without extratendinous soft tissue thickening and extensor tendonitis [32].

*Management*. Corticosteroid injections in the flexor synovial sheaths, NSIADs, and COX-2 inhibitors help to mitigate dactylitic pain and inflammation.

## 5. Arthralgia and Fibromyalgia

Arthralgia (noninflammatory joint pain) is more common than arthritis in IBD. Physical examination may reveal tenderness of the involved joints without evidence of inflammation. Fibromyalgia presents as generalized body pain, not limited to joint areas. Physical examination reveals generalized soft tissue tender points; the current diagnostic criteria are based on widespread pain index and symptom severity scoring [33]. 

*Management*. Acetaminophen and NSAIDs/COX-2 inhibitors are usually the first line therapy for arthralgia. Recently, there are reports about probiotics being useful in IBD patients with arthralgia. In an open label trial evaluating probiotic use in 16 IBD patients with arthralgia and no clinical or serological evidence of arthritis, an improvement in peripheral but not axial symptoms was noted using an articular index scoring [34]. Large controlled studies are needed to further clarify the role of probiotics in the management of arthralgia in IBD population.

Treatment of fibromyalgia is more complex and requires a multimodal approach.

Patient self-report measures such as symptoms severity score, visual analogue scores (VAS) for pain and fatigue, the health assessment questionnaire (HAQ), and the fibromyalgia impact questionnaire (FIQ) are useful to guide the therapy. Nonpharmacological interventions include patient education, graduated aerobic exercise program, cognitive behavioral therapy, and hydrotherapy. Antidepressants approved by FDA for treatment of fibromyalgia include duloxetine and milnacipran; tricyclic antidepressants like amitriptyline, although helpful, should be avoided in IBD patients due to their anticholinergic properties. Pregabalin and gabapentin may also be used with added benefit [35].

## 6. Isolated Sacroiliitis, Inflammatory Back Pain, and Ankylosing Spondylitis (AS)

Inflammatory back pain, the most common clinical feature of SpA, presents as insidious onset of pain, usually lasting for >3 months, associated with morning stiffness, and improves after activity. Isolated sacroiliitis is defined as radiologic evidence of inflammation of unilateral or bilateral sacroiliac joints in absence of clinical symptoms. Ankylosing spondylitis (AS), the prototypical SpA, is characterized by presence of inflammatory back pain with or without limitation of lumbar spine mobility along with radiological evidence of sacroiliitis. MRI has better sensitivity for diagnosing sacroiliitis as it can detect acute inflammation and marrow edema seen in early phase. Asymptomatic sacroiliitis has been seen in up to 16% of patients with IBD; with the use of MRI, however, the detection rate of sacroiliitis is now thought to be as high as 46%. Various criteria exist for definition of inflammatory back pain and AS. Classically the Calin criteria and the modified New York criteria have been most widely used [36, 37]. The Assessment of SpondyloArthritis international Society (ASAS) criteria, introduced in 2009, include the presence of sacroiliitis by imaging (radiography or MRI) plus at least one clinical feature (IBP, arthritis, enthesitis, dactylitis, uveitis, colitis, psoriasis, response to NSAID, family history of SpA, presence of HLA-B27, or elevated CRP) or presence of HLA-B27 and any two other clinical features. These criteria have sensitivity and specificity of 82.9% and 84.4%, respectively, and have been validated for diagnosing axial SpA in patients with low back pain [38].

*Management*. Education regarding the natural history of the disease and goal of the therapy is very important. Moderate aerobic exercise and physical therapy help alleviate pain, improve mobility, relieve symptoms, and maintain function.

NSAIDs and COX-2 inhibitors constitute the first line agents for symptom control in SpA. Naproxen (NPX) has been studied in patients with axial arthropathy and induces remission in about a third of the patients. IBD patients with only axial disease show marginal to no improvement with sulfasalazine and other disease modifying antirheumatic drugs (DMARD), such as MTX or azathioprine [39]. Sulfasalazine has shown to have no disease modifying effect on aggressive arthritis [15].

Introduction of anti-TNF*α* (ATA) agents has revolutionized the treatment of spondyloarthropathies. At the same time, they have emerged as the primary treatment options for moderate to severe IBD. They are further discussed under Biologic Treatment.

## 7. Biologic Treatment

### 7.1. Anti-TNF*α* (ATA) Therapies

ATA agents have been a major breakthrough in treatment of IBD as well as in the management of extra-articular manifestations of IBD [40]. TNF*α* is one of the prime molecules perpetuating inflammation in the gut-synovial axis [1]. ATA agents effectively neutralize the bioactivity of soluble TNF*α*; however that in isolation is unlikely to represent the only mechanism of action of anti-TNF*α* antibodies. Neutralization of soluble and transmembrane TNF*α*, restoration of mucosal integrity, inhibition of mucosal angiogenesis in gut mucosa, induction of T-cell apoptosis, apoptosis of transmembrane TNF*α* expressing cells, downregulation of costimulatory molecules like CD40, reduced production of proinflammatory cytokines including IL1, IL6, and IL8, and induction of T-cell population with a regulatory phenotype are other proposed mechanisms of action of ATA agents in CD [41].

Infliximab (IFX) is a mouse chimeric monoclonal antibody that targets the human TNF*α*; adalimumab (ADA) is a fully humanized IgG1 monoclonal antibody against the same. Etanercept (ETA) is a genetically engineered fusion protein consisting of two recombinant human TNF p75 receptors linked to an Fc portion of human IgG1 fragment. Golimumab (GOL) is a newer humanized monoclonal antibody against TNF*α*. Certolizumab (Fab fragment of humanized antibody against TNF*α*) selectively neutralizes human TNF*α* activity without inducing complement activation because of the absence of Fc fragment. Pegylated form of certolizumab (certolizumab pegol, CZP) allows for delayed metabolism and hence extended effect.

It is important to note that ATA agents are not similar in their clinical effects. Anti-TNF monoclonal antibodies (IFX, ADA) are useful for both axial and peripheral arthritis in CD patients [40]. IFX has been used mostly in CD patients with peripheral arthritis and has been found to be effective in controlling symptoms poorly responsive to conventional therapies [42]. TNF*α* receptor blocker ETA may benefit arthritis although it is not useful to control the bowel inflammation and may actually worsen it [43]. ADA is also thought to be useful in treatment of both AS and IBD, found to be effective in patients with predominantly peripheral arthritis [44].

IFX is currently the first line treatment for moderate to severe CD [45]. Long-term safety and efficacy of IFX in patients with SpA has been established beyond doubt [46]. IFX when used in combination with naproxen was twice as likely to achieve remission as compared to naproxen alone in patients with active axial SpA. Another study evaluating lower dose IFX (3 mg/kg q 8 weekly during 2nd year of the treatment instead of 5 mg/kg q6 weekly dose) in patients with active AS found successful results in suppressing the disease with lower dose IFX [47].

ADA has been found effective in induction as well as maintenance of remission in patients with moderate to severe CD including IFX nonresponders as well as those with predominant mucosal or fistulous lesions [48–50]. Multiple studies have evaluated safety and efficacy of ADA in treatment of axial arthritis in AS patient populations; the results of 5-year follow-up studies reveal continued benefit without loss of efficacy [51, 52]. Retention rates, frequencies of hospitalization, and overall effectiveness were found to be similar for IFX and ADA in CD patients [53]. ADA is used as 40 mg SC injections every other week for SpA and inflammatory arthritides; however, higher doses (160 mg SC on day 1, 80 mg SC on day 15, followed by maintenance dose of 40 mg SC every other week) are used for induction therapy of CD and possibly for IBD related arthritides. It is available as prefilled syringes and pens.

GOL, when administered subcutaneously, maintains remission in moderate to severe UC [54]. Treatment with GOL in AS patients has been found to be effective in preventing disease progression. Follow-up data up to 2 years showed promising safety and efficacy results [55]. GOL is commonly used as subcutaneous injections in doses to 50 mg SC q 4 weeks or 100 mg SC q 4 weeks. CZP has been USFDA approved for treatment and maintenance of remission in patients with moderate to severe CD [56]. CZP has been shown to reduce the signs and symptoms of axial spondyloarthropathies up to 24 weeks and the benefit is apparent as early as the first week. CZP is used as 400 mg SC injections every 4 weeks. Meta-analyses involving trials of all evaluated ATA agents suggest IFX, ADA, ETA, and GOL to be effective in reducing the signs and symptoms of the axial SpA [57]. In the patients of AS, the probability of obtaining remission with IFX, ADA, or ETA was not significantly different among all patients [29].

### 7.2. Other Therapies

Newer biologic molecules targeting pathways other than TNF*α* are promising options for patients not responding to ATA agents.

B cells responses leading to production of hTM-5 specific antibodies and subsequent complement mediated lysis of colonic epithelial cells underlie the humoral basis of autoimmunity in UC patients; some of the same mechanisms may contribute to joint inflammation and damage as per the gut-synovial hypothesis [1]. Rituximab (RTX) is a chimeric monoclonal antibody against B cell surface protein CD20. RTX when evaluated in SpA patients was found to have moderate efficacy in patients including SpA associated with CD; the response was more marked in patients who were ATA-naive [58].

Interaction between *α*4*β*7 integrin expressed on the surface of lymphocytes and addressin (also known as MadCAM-1) expressed on the high endothelial venules in Peyer's patches is considered to be a key event leading to homing and transmigration of the activated lymphocytes in the ileum of IBD patients [1]. Natalizumab is a humanized monoclonal antibody against *α*4 subunit of *α*4*β*7 and *α*4*β*1 integrins. Natalizumab has been shown to increase the rates of clinical remission and improve the quality of life and C-reactive protein levels in patients with moderate to severe CD [59]. In a pilot study, Natalizumab was also used in UC population [60]. Progressive multifocal leukoencephalopathy (PMLE) is a dreaded side effect of Natalizumab and limits its widespread use in clinical practice. Vedolizumab is a monoclonal antibody designed against the entire *α*4*β*7 heterodimer, thus making it more “gut-selective” with no reported cases of PMLE so far. Emerging evidence from randomized placebo controlled clinical trials suggests a potential role for vedolizumab in both CD [61] and UC [62], particularly in patients who have previously failed other biological therapy. These agents are expected to benefit inflammatory articular involvement as a similar interaction between integrins and cell adhesion molecules may exist at joints, resulting in synovial homing of activated lymphocytes.

Signaling via IL23/IL12 complex is one of the key events leading to transformation of naïve T cells to activated Th17 and Th1 cells [1]. Ustekinumab is a fully humanized monoclonal antibody against IL12/IL23 complex. It has been found to be effective as induction and maintenance therapy in moderate to severe CD patients resistant to ATA therapy [63]. Efficacy and safety of ustekinumab in patients with active psoriatic arthritis are well established [64]. In a recent prospective, open label trial in AS patients, ustekinumab was found to be effective in reducing of signs and symptoms in active AS.

## 8. Biosimilars

Biosimilars are biotherapeutic products designed to generate molecules similar to an already licensed reference product in terms of amino acid sequence, posttranslational modification, tertiary protein conformation, pharmacokinetics, receptor affinity, postreceptor effects, immunogenicity, safety, and efficacy [65]. Biosimilars can be seen as potentially cost saving products akin to generic versions of medicines. The availability of biosimilars as lower-cost alternatives of biologics must be carefully weighed against possible issues of safety and efficacy [66]. The infliximab biosimilar CT-P13 introduced in June 2013 is the first biosimilar monoclonal antibody registered for the treatment of IBD. Interestingly, the same molecule was evaluated in patients with AS in a prospective, double blind placebo controlled trial and was found to have pharmacokinetic, safety, and efficacy profile similar to IFX. The same agent is also being evaluated in RA patients [67].  Table 3 lists various biosimilars that are currently being investigated in patients with IBD or inflammatory arthritis. Biosimilars for adalimumab, rituximab, and etanercept are in various stages of clinical trials and are expected to populate the market in the coming decade [68].

## 9. Conclusion

Peripheral arthritides as well as axial involvement similar to SpA are common in IBD. Joint inflammation commonly runs a parallel clinical course to gut inflammation; consequently there are considerable overlaps in the treatment of both. Use of ATA agents is the cornerstone of the anti-inflammatory therapies; newer agents including biosimilars are on the horizon.

## Figures and Tables

**Table 1:** Groups of extraintestinal manifestations by relationship to gut inflammation.

Group 1 EIMs that run a course parallel to gut inflammation	Group 2 EIMs that run a course independent of gut inflammation	Group 3 EIMs that may or may not be related to gut inflammation
Type 1 peripheral arthritis	Type 2 peripheral arthritis	Pyoderma gangrenosum
Aphthous ulcers	Ankylosing spondylitis	Primary sclerosing cholangitis
Erythema nodosum	Uveitis	
Episcleritis	Orbital myositis	
	Gastrocnemius myalgia syndrome	

Table is reproduced from Sheth et al. [1] with permission from Lippincott Williams and Wilkins.

**Table 2:** Musculoskeletal manifestations in IBD.

	Salient features	Prevalence
Peripheral arthropathies		

Type 1 peripheral arthritis	Oligoarticular Asymmetric Large joints of lower extremities Self-limiting Nonerosive Parallels disease activity Associated with HLA-B27 carriage	3.6–6%
Type 2 peripheral arthritis	Polyarticular Symmetric Small joints of upper extremities Progressive Erosive Independent of disease activity No association with HLA-B27	2.5–4%
Arthralgia without arthritis	Pain without swelling or erythema	5.3–16%
Enthesitis	Pain and swelling at the tendon insertion site	6–50%
Dactylitis	Pain and swelling of the entire digit	2–4%

Axial arthropathies		

Inflammatory back pain	Insidious onset Back pain lasting > 3 months After periods of inactivity Associated with stiffness No associated radiological findings	17–22%
Isolated sacroiliitis	Imaging studies showing erosion, or sclerosis of the sacroiliac joints May be asymptomatic HLA-B27 negative	16–46%
Ankylosing spondylitis	Combination of inflammatory back pain and imaging studies showing bilateral sacroiliitis grade ≥ 2 or unilateral sacroiliitis grades 3-4	1–11.4%

Other		

Fibromyalgia syndrome	Generalized body pain for ≥3 months 11 out of 18 tender points	3–3.7%
Osteopenia	BMD *T*-score ≤ −1.0	32–36%
Osteoporosis	BMD *T*-score ≤ −2.5	7–15%
Osteonecrosis	Marrow infarction Most common site head of femur	<0.5%
Myopathy	Multifactorial in etiology	Rare
Orbital myositis	Localized inflammation of extra-ocular muscles	Rare
Gastrocnemius myalgia syndrome	Calf pain as presenting complaint Gastrocnemius muscle involvement	Rare

BMD: bone mineral density.

Table is reproduced from Sheth et al. [1] with permission from Lippincott Williams and Wilkins.

**Table 3:** Ongoing clinical trials with biosimilars in participants with IBD or inflammatory arthritis.

Parent drug	Biosimilar agent	Sponsor	Condition	Trial description	ClinicalTrials.gov identifier	Status	Phase
IFX	CT-P13	Celltrion	AS	An extension study to demonstrate the equivalence of long-term efficacy and safety of CT-P13 in patients with ankylosing spondylitis who were treated with infliximab (Remicade or CT-P13) in study CT-P13 1.1	NCT01571206	COM	1

IFX	CT-P13	Celltrion	RA	An extension study to demonstrate long-term efficacy and safety of CT-P13 when coadministered with methotrexate in patient With rheumatoid arthritis who were treated with infliximab (Remicade or CT-P13) in study CT-P13 3.1	NCT01571219	COM	3

IFX	CT-P13	Celltrion	CD	Demonstrate noninferiority in efficacy and to assess safety of CT-P13 in patients with active Crohn's disease	NCT02096861	REC	3

IFX	CT-P13	Celltrion	AS	Program evaLuating the Autoimmune disease iNvEstigational drug cT-p13 in AS patients (PLANETAS)	NCT01220518	COM	1

IFX	CT-P13	Celltrion	RA	Program evaLuating the Autoimmune disease iNvEstigational drug cT-p13 in RA patients (PLANETRA)	NCT01217086	COM	3

IFX	IFX biosimilar	Diakonhjemmet Hospital	RA AS PsA UC CD	A randomized, double-blind, parallel-group study to evaluate the safety and efficacy of switching from innovator infliximab to biosimilar infliximab compared with continued treatment with innovator infliximab in patients with rheumatoid arthritis, spondyloarthritis, psoriatic arthritis, ulcerative colitis, Crohn's disease and chronic plaque psoriasis the nor-switch study	NCT02148640	REC	4

IFX	SB-2	Samsung Bioepis	RA	A study comparing SB2 to Remicade in subjects with moderate to severe rheumatoid arthritis despite methotrexate therapy	NCT01936181	ONG	3

ETA	SB-4	Samsung Bioepis	RA	A study comparing SB4 to Enbrel in subjects with moderate to severe rheumatoid arthritis despite methotrexate therapy	NCT01895309	ONG	3

ETA	CHS-0214	Coherus	RA	Comparison of CHS-0214 to Enbrel (etanercept) in patients with rheumatoid arthritis (RA) (CHS-0214-02)	NCT02115750	REC	3

RTX	CT-P10	Celltrion	RA	Demonstrate the equivalence of CT-P10 to MabThera with respect to the pharmacokinetic profile in patients with rheumatoid arthritis (Triad RA)	NCT01534884	ONG	1

RTX	BCD-020	Biocad	RA	Study of safety and efficacy of BCD-020 comparing to MabThera in patients with rheumatoid arthritis (BIORA)	NCT01759030	ONG	3

RTX	CT-P10	Celltrion	RA	Long-term efficacy and safety of CT-P10 in patients with RA	NCT01873443	ONG	1

Data are from http://clinicaltrials.gov/.

Data are current as of April 17, 2015.

IFX: infliximab, ETA: etanercept, RTA: rituximab, RA: rheumatoid arthritis, AS: ankylosing spondylitis, CD: Crohn's disease, PsA: psoriatic arthritis.

Trial status: COM completed, REC recruiting, ONG trial is ongoing, but not recruiting participants; NYR trial is registered but not yet recruiting participants.
